# The link between chronic pain and Alzheimer’s disease

**DOI:** 10.1186/s12974-019-1608-z

**Published:** 2019-11-06

**Authors:** Song Cao, Daniel W. Fisher, Tain Yu, Hongxin Dong

**Affiliations:** 1 Department of Pain Medicine, Affiliated Hospital of Zunyi Medical University, 149 Dalian Street, Zunyi, 56300 Guizhou China; 2 Guizhou Key Lab of Anesthesia and Organ Protection, Affiliated Hospital of Zunyi Medical University, 149 Dalian Street, Zunyi, 56300 Guizhou China; 3 Department of Psychiatry and Behavioral Sciences, Northwestern University Feinberg School of Medicine, 303 East Chicago Avenue, Chicago, IL 60611 USA

**Keywords:** Chronic pain, Alzheimer’s disease, Locus coeruleus, Norepinephrine, Noradrenergic system, Microglia, Prefrontal cortex

## Abstract

Chronic pain often occurs in the elderly, particularly in the patients with neurodegenerative disorders such as Alzheimer’s disease (AD). Although studies indicate that chronic pain correlates with cognitive decline, it is unclear whether chronic pain accelerates AD pathogenesis. In this review, we provide evidence that supports a link between chronic pain and AD and discuss potential mechanisms underlying this connection based on currently available literature from human and animal studies. Specifically, we describe two intertwined processes, locus coeruleus noradrenergic system dysfunction and neuroinflammation resulting from microglial pro-inflammatory activation in brain areas mediating the affective component of pain and cognition that have been found to influence both chronic pain and AD. These represent a pathological overlap that likely leads chronic pain to accelerate AD pathogenesis. Further, we discuss potential therapeutic interventions targeting noradrenergic dysfunction and microglial activation that may improve patient outcomes for those with chronic pain and AD.

## Introduction

Chronic pain, defined as pain lasting more than 3 months, is very common in the elderly and is a significant issue in both individual clinical practices and the healthcare system. The prevalence of chronic pain in older people living in the community is reported to be 38.5% [1], which is higher than that in the adult population at large (19%) [2] and schoolchildren (6%) [3]. Though chronic pain is often understood as aberrations in sensory processes, it is also highly associated with cognitive, emotional, and social dysfunction [4]. In contrast to acute pain, cognitive and emotional deficits seem to be particularly prominent when pain turns chronic, and the many failed treatments that solely target peripheral mechanisms of pain underly the increased complexity of pain perception with chronicity [5, 6]. Dysfunctions in pain perception mediated by the central nervous system (CNS) are likely to play a key role in the process of pain chronification, and central sensitization in pain processing pathways influences cognitive and emotional processing, which has been supported by both human [7, 8] and animal work [9, 10]. Together, a bidirectional interaction likely exists between chronic pain and cognitive deficits.

Alzheimer’s disease (AD), a neurodegenerative disorder and most common form of dementia, characterized by cognitive and behavioral impairments, is often co-morbid with chronic pain. The reported prevalence of chronic pain in AD patients was 45.8%, based on a recent meta-analysis [11]. In fact, pain may be underestimated in AD patients as they may be unable to communicate their pain and request attention as effectively as their cognitively normal peers [12]. Importantly, pain is observed more prevalently in patients with severe dementia [13], and intensity of pain is also positively correlated with dementia severity [14–16]. Though a bidirectional correlation exists between chronic pain and AD, a clear mechanistic link remains elusive. Growing evidence suggests patients with chronic pain or AD share some common pathologies, including abnormalities of the noradrenergic system in the locus coeruleus (LC) [17], activation of microglia in brain areas such as the frontal cortex, and increased central neuroinflammation in these regions [18]. The LC extends axons that innervates most brain area and is the main center of norepinephrine (NE) synthesis and subsequent neurotransmission in the CNS. Interestingly, it has been suggested that changes in LC-NE signaling contributes to microglial dysfunction [19].

In this review, we provide current evidence for the link between chronic pain and AD and the overlapping pathological changes in these two disorders. Additionally, we discuss the possibility that chronic pain aggravates AD pathogenesis through dysfunction of LC-NE signaling and subsequent microglial activation-induced neuroinflammation.

## The link between chronic pain, cognitive impairment, and dementia

It has been reported that chronic pain is associated with increased self-rated and objective cognitive deficits [15, 20]. These cognitive deficits are not specific to a particular pain modality and can be observed in fibromyalgia [21], postherpetic neuralgia [22], and chronic back pain [23], to name a few. Epidemiological analyses of community-dwelling residents and pain clinics estimate that at least 50% of people living with pain report cognitive problems [24], and a similar proportion demonstrate impairment on objective cognitive tests [25]. Further, clinical observations indicate that the intensity of pain is positively correlated with the degree of cognitive impairment [10, 26, 27].

Chronic pain is also associated with dementia. In the Einstein Aging Study, one of the longest running prospective cohort studies of aging, 1114 elderly participants [28] were assessed and 114 (10%) of the subjects developed dementia over 4.4 years [29]. In this study, higher levels of pain interference, defined as the degree of pain-related impairment in activities of daily living, were associated with a higher probability of developing dementia [29]. However, it is reported that there was no relationship between the intensity of pain and developing dementia [29], which may be due to (1) dementia patients and their caregivers routinely underreport pain intensity, (2) chronic pain may manifest differently in dementia patients, and (3) more severe cognitive impairments leading to reduced ability to articulate pain concerns [30–32]. Another study [33] also supported pain interference being significantly and positively associated with AD and related dementia (ADRD). In this cohort of 25,009 older adults (> 65 years), those with pain interference either with or without osteoarthritis had significantly higher odds of ADRD relative to those without osteoarthritis or pain interference [33]. Overall, these studies suggest that chronic pain, especially in patients with pain interference, may hasten dementia pathogenesis in addition to its effects on subclinical cognitive dysfunction.

Another similarity is that chronic pain and AD display abnormalities of gray matter volume, and neuroimaging suggests that cognitive decline in patients with chronic pain may be related to gray matter volume changes in the brain [4]. Many of these altered brain areas are involved in sensory perception, the affective component of pain, and cognition [34, 35]. For instance, gray matter volume loss has been found in the amygdala, entorhinal cortex, parahippocamal gyrus, anterior cingulate cortex, thalamus, and insula [36–38]. Additionally, reduced gray matter volume in brain areas involved in cognitive function, such as the dorsolateral prefrontal cortex (dlPFC) [39], medial prefrontal cortex (mPFC) [37], and hippocampus [40]. We found that postherpetic neuralgia patients displayed decreased gray matter volume in the frontal lobe compared with healthy controls or otherwise healthy herpes zoster patients [41]. Interestingly, some of these areas are among earliest sites of degeneration in AD [42], and cortical gray matter volume is correlated with cognitive decline in AD [43]. Though different pathological mechanisms may exist and independently contribute to gray matter atrophy in chronic pain and AD, an interaction may be present in these two disease states that synergistically increases neurodegeneration and cognitive decline.

To date, a considerable number of animal experiments have investigated the cellular and molecular mechanisms between chronic pain and cognitive deficits [44, 45]; however, few studies have focused on whether a common mechanism underlies cognitive decline in co-morbid chronic pain and AD. In one notable study [45], chronic inflammatory pain accelerated cognitive impairment in 5-month old APP/PS1 mice, a prominent AD model, but not in wildtype animals. As APP/PS1 mice rarely develop overt cognitive deficits before 9–12 months of age, the development of memory impairment in this study suggests that chronic pain accelerates AD pathogenesis and subsequent cognitive decline. While this study suggests animal models may be useful for investigating a mechanistic relationship between AD and chronic pain, this investigation still needs to be done.

## Chronic pain induces dysfunction of the LC-NE system and pro-inflammatory microglial activation

The LC has been implicated in a variety of physiological functions including attention, memory, emotion, stress reactions, and pain modulation [46]. The LC is located in the dorsal pontine nucleus and provides descending noradrenergic input to the spinal cord, forming the LC-spinal descending pain modulation system. It also extensively projects to most regions of the brain, particularly the frontal cortices and the limbic system [47], and is well known as the predominant source of the neurotransmitter NE in the brain [48].

### Chronic pain induces dysfunction of LC-NE system

Dysfunction of LC-NE system associated with chronic pain has been reported mostly in animals. Significant increases in the expression of tyrosine hydroxylase, dopamine beta-hydroxylase (DβH), the NE transporter, the α_2_-adrenoceptor, and burst firing have been seen in the LC of rats with a chronic constriction injury, a widely used neuropathic pain model [47, 49]. This results in more NE release [50, 51], although these changes were only evident when pain became chronic (28-day pain duration) and not during the acute period (7-day pain duration) [47, 49]. Although most of the animal studies reported that the LC is activated in chronic pain, one study showed opposite results [52]. In particular, in streptozocin-treated rats, a model of diabetic neuropathy, LC firing activity and expression levels of tyrosine hydroxylase, pCREB, and the NE transporter were reduced in the LC when anxiety-like and depression-like behaviors were observed, a time point considered to be representative of chronic pain [52]. The distinct nociceptive sensitivity time-courses as well as the LC functions between the chronic constriction injury and streptozocin models indicate that specific neuroplastic mechanisms depend on pain modality [52], which adds a layer of complexity in determining how chronic pain may be affected by LC-NE neurotransmission.

Changes in LC function and plasticity will affect NE transmission in numerous brain regions, and it is very possible that changes in LC-NE function are not uniform but instead vary based on downstream targets. For example, abnormally increased LC-PFC neurotransmission has been widely reported in animals with chronic pain and leads to increased noradrenergic fiber sprouting and intrinsic excitability in the PFC, mediated in part by α_2_-adrenoreceptors and HCN channels [53]. This increase in plasticity in the PFC coincides with shifting NE neurotransmission in other specific brain areas innervated by the LC [54]. Despite this increase in noradrenergic markers, microdialysis techniques showed that 28 days after chronic constriction injury, no significant difference was found in basal NE release in the LC and PFC in rats [49]. However, another study showed that in the PFC, the sensitivity of α_2_-adrenoceptors is enhanced, and 6 weeks after spinal nerve ligation, the NE content of the PFC was augmented significantly, coupled with impaired attention [50]. These data suggest that pain duration is an important factor for the neuroplastic LC changes and also suggest that changes in LC-NE neurotransmission is likely more nuanced than those determined by measuring basal neurotransmission alone [47].

Overall, chronic pain-induced LC-NE system dysfunction during chronic pain is complex and likely depends on the chronic pain duration, modality, and downstream neuron function and location. However, in terms of brain regions that are affected by cognition, such as the PFC, enhanced neurotransmission of NE results in increased excitability that coincides with aberrant cognitive and emotional behaviors.

### Chronic pain induces microglial activation and neuroinflammation

Microglia are the primary innate immune cells in the CNS, which is otherwise relatively immunoisolated [55]. As one of the constituent cells of blood-brain barrier, microglia express many kinds of receptors which recognize exogenous or endogenous insults to the CNS and initiate an immune response [56]. For example, it has been reported that microglia control the spread of neurotropic virus infection [57], which can induce encephalitis and neurodegeneration [58].

Microglial activation includes pro-inflammatory activation and anti-inflammatory activation [59, 60]. Microglial pro-inflammatory activation promote neuropsychiatric disease through release of pro-inflammatory molecules such as tumor necrosis factor-α (TNF-α), interleukin (IL)-1β, IL-6, inducible nitrous oxide synthase (iNOS), and reactive oxygen species [60]. Activated microglia due to chronic pain may play critical roles in pain occurrence and maintenance [61]. In chronic pain, neuroinflammation driven by microglia is a characteristic feature [62]. In fact, microglia are initiators of a postinjury, neuroimmune response that contributes to the transition from acute to chronic pain [63]. In the chronic pain state, pro-inflammatory microglia release cytokines and chemokines associated with inflammation, such as IL-6, IL-1β, and TNF-α [64, 65]. This pro-inflammatory state leads to changes in synaptic remodeling, brain connectivity, and network function [66].

Chronic pain-induced microglial activation and neuroinflammation have been reported in both humans and animal models. In one study using PET-MRI, patients with chronic lower back pain showed increased microglia and astrocyte activation in the thalamus and the putative somatosensory representations of the lumbar spine and leg, as evidenced by elevated signal of translocator protein, a marker of microglia and astrocytes [67]. In another study, by using [^11^C]PBR28 PET, microglial activation signal was widely elevated in certain cortical regions of patients with fibromyalgia, a condition typified by chronic, neuropathic pain [68].

Additionally, a large number of animal studies have shown that chronic pain increases microglial pro-inflammatory activation and neuroinflammation in the brain. Specifically, microglial activation has been detected in the PFC [64, 69, 70], hippocampus [71], anterior cingulate cortex [72], amygdala, nucleus accumbens, thalamus, and sensory cortex [64, 73, 74] in different kinds of chronic pain animal models [75]. For instance, rats with spared nerve injury showed microglial activation in the PFC as evidenced by increased expression of CD68, iNOS, IL-1β, IL-6, TNF-α, and 8-OH-dG [70, 74]. Spared nerve injury mice also displayed TNF-α upregulation in bilateral hippocampi [65]. Similarly, rats with spinal nerve ligation showed microglial activation in the PFC [69], and mice with chronic constriction injury developed increased microglial inflammation in the mPFC, hippocampus, and amygdala evidenced by increased CD11b and TNF-α expression [64].

In all, chronic pain is associated with upregulation of pro-inflammatory microglia in varied brain areas, including those responsible for cognition and emotion. Microglial activation induces neuronal dysfunction, such as the LC-NE dysfunction, which may feed forward to enhance further microglia-driven neuroinflammation.

## Chronic pain may aggravate AD neuropathogenesis through LC-NE-induced microglial neuroinflammation

Chronic pain and AD brains not only display abnormal LC structure and function but also dynamic changes in NE turnover in LC-projecting areas [47, 76]. Although the shifts of NE content may not perfectly overlap in all brain areas in these two disease states, LC-NE pathological changes in select regions could be one of the initiators that leads to a final common outcome: pro-inflammatory activation of microglia and neuronal dysfunction.

### Dysfunction of the LC-NE system in AD brain

Similar to chronic pain, the function and structure of the LC are disrupted in AD, and the LC is one of the earliest brain regions affected during AD development and progression [77]. A loss of LC-noradrenergic neurons is observed in autopsy specimens of AD patients, with estimated 50% [78] to 60% [79] LC cell loss, which is more dramatic than the 41% neuron loss observed in the PFC [80]. However, there is evidence that axonal sprouting and dendritic arborization increases, perhaps as compensation for the loss of cells [81]. Concomitantly, increased activity of surviving LC neurons in AD patients is also reported [82, 83], and post-mortem autoradiography detected significantly decreased NE transporter in the human AD brain, again suggesting compensatory mechanisms adjusted for cell loss [84].

Due to the complexity in these degenerative and compensatory mechanisms, it is not surprising that there are conflicting reports of the levels of NE in various brain areas in AD patients. Some reported a decrease in NE and some demonstrated that NE levels in AD patients remain constant or even elevated in the cerebrospinal fluid [76, 78, 85]. These differences in NE neurotransmission across studies may also reflect different points in AD pathogenesis. Therefore, although neuron loss occurs in the LC, NE content does not necessarily decrease in the AD brain, as the system likely compensates by increasing LC excitability and decreasing NE reuptake, and increased NE early in the disease may further exacerbate neurodegeneration. Although noradrenergic neuron loss in the LC correlates significantly with the duration of AD [86], NE concentration decreases were found in brain regions associated with cognitive deficits, namely the midtemporal cortex and the orbital-frontal cortex [78]. Mirroring the loss of LC neurons in humans, animal models of AD also demonstrated significant aberrations in the LC-NE system. For instance, the APP/PS1 mouse has demonstrated degenerated noradrenergic neurons and fibers in the LC [87]. Further, hyperphosphorylated tau causes reduced NE neurotransmission from the LC to the forebrain and certain subcortical areas, such as the hippocampus, suggesting that tau may be one of the AD-related mediators of LC dysfunction [88]. These studies suggest that patients with AD have a progressive decrease in LC neurons, and part of the clinical symptoms of AD may be due to reduced noradrenergic neurotransmission mediated by LC degeneration, especially in mid- and late-stages of disease progression [89].

### NE turnover influences microglial activation and neuroinflammation

Homeostasis of the LC-NE system is important for controlling central inflammation because numerous studies have shown that NE can effectively inhibit inflammation, including microglia-related neuroinflammation [19]. On the other hand, recent studies indicate that NE could be pro-inflammatory [90]. Therefore, when the homeostasis of LC-NE system is destroyed, either excessive or insufficient NE may lead to neuroinflammation.

Microglia are well equipped to respond to NE signaling by expressing the noradrenergic α_1A_, α_2A_, β_1_, and β_2_ receptors [91, 92] and strong interactions exist between NE and microglia [87, 92]. Some evidence supports that pro-inflammatory activation of microglia may be due to activation of the LC and abnormal and excessive release of NE [90]. NE enhances Aβ-mediated IL-1β secretion through action at β-adrenoceptors in THP-1 cells [93]. NE also increases COX-2 and prostaglandin E2 production induced by LPS via β-adrenoreceptors in rat primary microglia [94]. NE plays both roles as a facilitator or a suppressor for microglial pro-inflammatory reactions via activating cAMP and modulating downstream MAPK and NF-κB signaling [95]. Therefore, chronic pain-induced LC-NE neuron hyperactivity and increased supply of NE to brain areas such as PFC may result in microglial pro-inflammatory activation and exacerbate neuroinflammation in these areas in AD [64, 70].

However, more evidence indicates that NE inhibits microglial activation and suppresses the production of pro-inflammatory factors such as IL-6 and TNF-α [91]. Studies suggested that NE dampens microglial reactivity [96], as NE negatively regulated the transcription of inflammatory genes encoding pro-inflammatory cytokines and chemokines in microglia [97, 98], likely through direct action on adrenergic receptors on these cells [92]. For instance, noradrenergic depletion with DSP4 treatment increased microglial activation and the expression of iNOS and COX2 in the hippocampus and the frontal cortex of aged APP V717 transgenic mice [87]. Co-activation of β1 and β_2_ in hippocampal slice cultures reduced microglial activation from a pro-inflammatory LPS plus oxygen-glucose deprivation insult and resulted in an overall reduction in TNF-α, IL-6, and MCP-1 [99]. Similarly, β-adrenergic agonism in microglia co-cultured with cortical neurons protected these cells from death by downregulating TNF-α, IL-6, and free radical expression [100, 101]. In line with the dampening of microglial activation, catecholamines also inhibit nitric oxide production from microglia, perhaps by causing a decrease in iNOS [101].

## Activated microglia and neuroinflammation promote AD pathogenesis

### Microglial activation and neuroinflammation increase in AD

Recently, inflammation-associated PET studies demonstrated microglial activation in the brains of AD patients. In addition, microglial activation was found occurring before cognitive decline in AD patients, suggesting that it may be an early precipitant of AD progression [102]. By using PET-MRI in AD patients, two peaks of microglial activation were detected during the trajectory of AD pathogenesis, which may represent an early protective peak and a later pro-inflammatory peak [103].

Microglial activation and neuroinflammation are found in the brains of AD animal models. For example, microglia show increased proliferation in well-characterized mouse models of AD, including APP/PS1, 5XFAD, and APP23 mice [104, 105], and increased expression of pro-inflammatory markers such as CD36, CD14, CD11c, MHC-II, and iNOS [106, 107].

Microglial activation may play a dual role in AD. At the early stage of AD, activated microglia display mainly anti-inflammatory phenotypes [108], while chronic activation of microglia contributes to neurotoxicity and induces synapse loss by triggering pro-inflammatory cascades [109, 110]. Similar to microglial activation in chronic pain, loss of microglial homeostatic functioning and subsequent transition to prolonged and pathological neuroinflammation exists in AD patients [109, 110]. Despite the complexity in the role of microglia in AD pathogenesis, studies suggest that during AD progression, microglia predominantly support pro-inflammatory processes and promote cognitive decline. In fact, reactivations of microglia and neuroinflammation are now considered characteristics of AD pathogenesis [111].

### Microglial activation and neuroinflammation hastens AD pathogenesis via Aβ

Microglia are found clustered around amyloid plaques in both humans [112] and AD mice [113] and have been shown to regulate plaque dynamics [114]. Under non-pathological conditions, microglia play an important role in regulating Aβ deposition [115], and abundant evidence suggests that properly functioning microglia are involved in the clearance of Aβ and limiting the expansion of plaques [116, 117]. With early exposure to Aβ, activated microglia may phagocytose toxic Aβ and produce survival-promoting trophic factors in the AD brain [118]. However, some studies show that microglia become activated with prolonged exposure to Aβ and will undergo a pro-inflammatory response [114, 119], resulting in the secretion of synaptotoxic/neurotoxic cytokines, chemokines, and reactive oxygen/nitrogen species [118, 120, 121]. This is highlighted by studies in post-mortem AD brains and mouse models, where prolonged Aβ deposition leads to alterations in microglia, such as P2X7 receptor upregulation and activation of the innate immune response characterized by release of pro-inflammatory cytokines, acute phase proteins, and complement components [122–124] that cause microglia-mediated synapse and neuron loss [125].

In addition, microglia may release large amounts of fibrillar Aβ and can promote Aβ plaque formation [126]. In addition to directly mediating neurodegeneration, microglia-derived ASC specks may cross-seed Aβ in AD, causing progression of the proteinopathy and spread of pathogenesis across the brain [127]. Although the relationship between microglia and Aβ is complex, the sustained presentation of Aβ seems to promote pro-inflammatory activation of microglia that ultimately furthers the synapse loss that is so highly correlated with cognitive decline.

### Microglial activation aggravates AD via tau pathology

Neurofibrillary tangles in the brain of AD patients increase in parallel with colocalized expression of microglial pro-inflammatory activation and tau kinases [128]. Hyperphosphorylated tau, misfolded tau, and truncated tau co-occurs with microglia proliferation and increased expression of inflammatory genes such as *Aif1* (encoding IBA-1), *Ptgs2* (encodes COX2), *IL-1β*, *IL-6*, and *Tnf-α* in the LC [128]. These findings suggest that activated microglia may participate in driving tau pathology in AD.

It has been extensively reported that trans-synaptic propagation of tau occurs through anatomically connected synapses; however, microglia are implicated in spreading tau [129] by endocytic phagocytosis and exocytic release of exosomes in pathways independent of synaptic transmission [130]. Microglial uptake and exosomal release of tau may play a key role for tau spreading between cells in the brain [130]. In vitro, microglia isolated from human AD cases and rTg4510 tauopathy mice are capable releasing tau seeds [129]. These microglia took up tau in the conditioned media but cannot entirely neutralize its seeding activity. These data suggest that microglia only have limited capacity to take up and break down seed competent tau, and inefficiency in this process may play a role in the spread of tau pathology [129]. In addition, evidence indicates that reactive microglia are sufficient to drive tau pathology and are highly correlated with the spread of pathological tau in the brain [131], while depletion of microglia suppresses tau propagation [130]. In transgenic tau mouse models, when microglia were activated by deleting CX3CR1, tau pathology was increased [131, 132]. These studies suggest that microglial activation contributes to tau pathology during AD pathogenesis.

## Therapeutic implications of targeting the LC-NE system and microglia in co-morbid chronic pain and AD

### Increasing NE

Maintaining the balance of the LC-NE system may help the prevention and treatment of AD. Animal studies suggested that modulation of microglial activation by increasing NE level would be one approach for AD alleviation [133]. NE could be neuroprotective against Aβ toxicity through redox cycling and reduction of intracellular oxidative stress [134]. The NE precursor L-threo-3,4-dihydroxyphenylserine (L-DOPS) rescued spatial memory deficits in DBH^−/−^ AD mice [135]. It is also reported that L-DOPS restored the balance of inflammation, facilitated microglia migration and Aβ phagocytosis, and reversed learning deficits in DSP4-induced LC lesion AD mice [87]. Thus, increasing NE concentrations may facilitate anti-inflammatory functions of microglia in AD and promote microglial migration and phagocytosis of Aβ.

Although aforementioned animal studies showed that increasing NE levels can improve AD symptoms, the efficacy of this kind of treatment still needs to be tested clinically. In a clinical trial in AD patients, atomoxetine, a selective noradrenergic reuptake blocker, did not improve cognitive function [136]. However, as the relative NE levels and downstream pathological changes are varied based on disease stage and brain location, increased NE may result in microglial activation and inflammation priming if it occurs during certain phases of AD pathogenesis [137], and increased NE is even suspected to be the etiological factor of AD [138]. Addressing this issue will help to judge the feasibility of NE therapy during different stages of AD pathogenesis. Meanwhile, if AD patients have co-morbid chronic pain, it needs to be realized that LC-NE system dysfunction is likely the result of a combination of the two disease processes. Patients with AD have a decrease in noradrenergic neuron in the LC, although some studies suggest that the remaining noradrenergic neurons can compensate for this cell loss by increasing their activity and may even increase NE neurotransmission in the AD brain during certain stages of the disease [83, 138]. A dichotomy may emerge between AD patients with co-morbid chronic pain and those without: in the AD patient without chronic pain, there may be a relative deficiency of NE in brain regions, especially when compensatory mechanisms have been exhausted, and increasing NE supply in these brain areas may be beneficial; however, in AD patients with chronic pain, NE neurotransmission may be enhanced in brain areas such as the mPFC and hippocampus, which could possibly result in microglial pro-inflammatory activation [70]. Therefore, in AD patients with chronic pain, supplementation of NE via reuptake inhibitors may paradoxically exacerbate AD pathogenesis.

### Inhibiting microglial activation

Another possible way to prevent and relieve AD is to directly inhibit pathological activation of microglia. Minocycline, a semisynthetic tetracycline derivative, is widely used to inhibit microglial activation [139]. In AD animals, minocycline reduced AD symptoms by reducing neuroinflammation, CNS pathology, and preventing cell death [140, 141]. For instance, in APP/PS mice, minocycline increased the survival of new dentate granule cells and improved behavioral performance in a hippocampus-dependent learning task [142]. In Tg2576 mice, minocycline attenuated deficits in learning and memory in Aβ-infused rats [140]. Clinical trials showed that minocycline is effective to treat chronic pain such as peripheral and autonomic neuropathies in type 2 diabetic patients [143], rheumatoid arthritis [144], and affective pain evaluated by McGill Pain Questionnaire in a cohort of patients with neuropathic pain, although the pain intensities did not change [145]. These data suggest that minocycline can reduce chronic pain and microglial activation in AD patient. However, the efficacy of minocycline in AD patients remains to be revealed, although one clinical trial is evaluating minocycline’s efficacy in patients with mild cognitive impairment or AD (NCT01463384).

## Conclusions and future directions

**Fig. 1:**
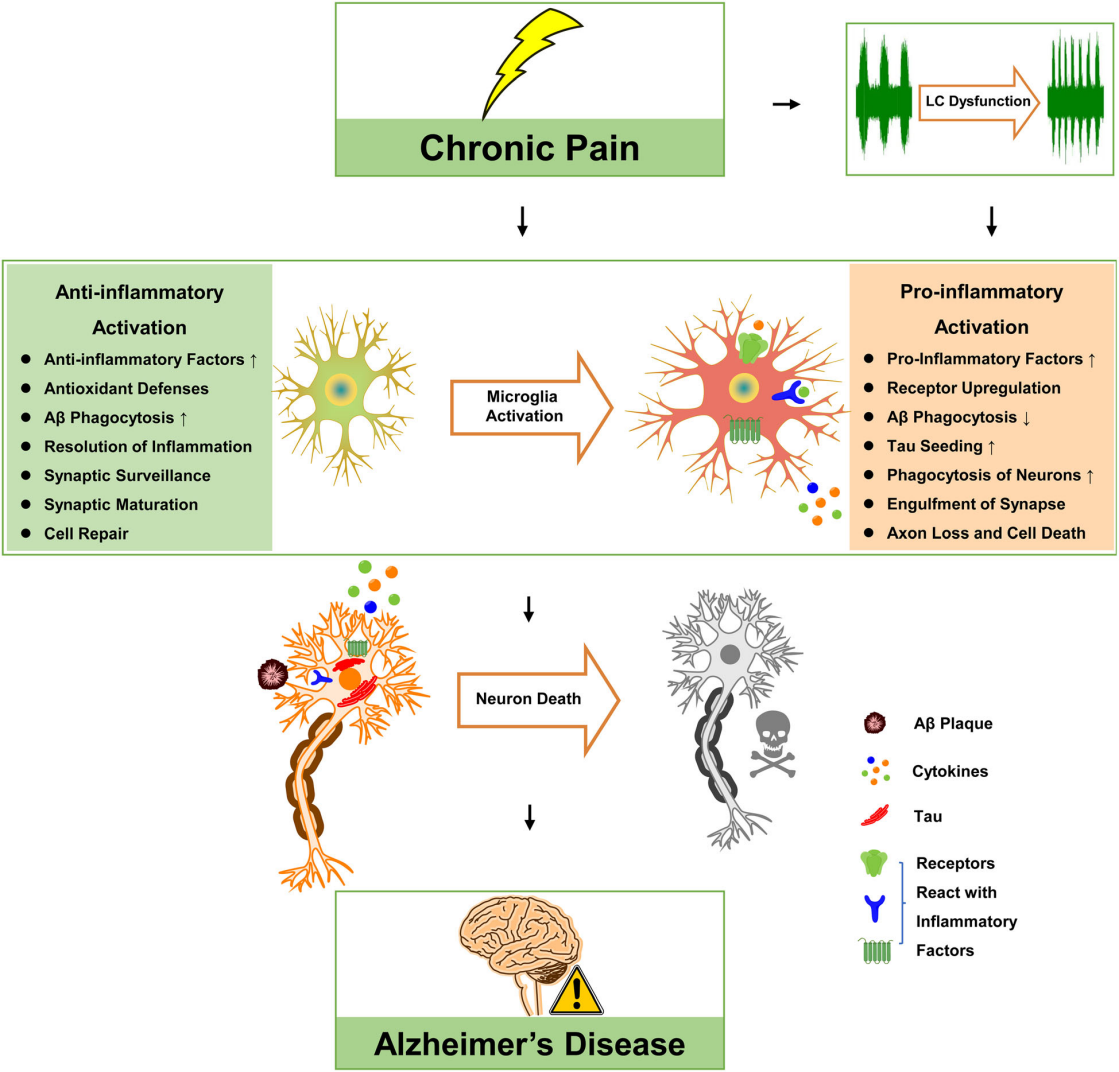
Illustration depicting a possible mechanism of chronic pain induced Alzheimer’s disease pathogenesis through locus coeruleus (LC)-noradrenaline (NE) system dysfunction and microglial neuroinflammation. Chronic pain induces pathological activation of LC-NE system and result in an increase of NE release in brain areas such as the prefrontal cortex and hippocampus, which could be one of the mechanisms of chronic pain-induced microglial pro-inflammatory activation. Pro-inflammatory activation may exacerbate AD pathogenesis via decreased Aβ phagocytosis, increased tau seeding, loss of synaptic function, and cytokine-induced neuron death in these brain regions

This review discusses the link between chronic pain and AD and a potential mechanism underlying this connection. Dysfunction of LC-NE system that may trigger pro-inflammatory activation of microglia in chronic pain could be one of the bridges between chronic pain and AD aggravation (Fig. 1).

Though chronic pain-induced LC-NE dysfunction may aggravate AD pathogenesis through pro-inflammatory microglia, the pattern of LC-NE dysfunction in the co-morbid AD and chronic pain state is not elucidated. Whether chronic pain induces neuronal loss in the LC has not been reported yet, but it is possible, especially with long-lasting pain (i.e., > 3 months). Studies to examine whether chronic pain can induce or aggravate cognitive deficits as well as behavioral and psychiatric symptoms in aged and AD models would be helpful in beginning to confirm a causal relationship, and tracking AD-related biomarkers and pro-inflammatory factors released from activated microglia in brain regions related to AD pathogenesis, such as the PFC and hippocampus, would further help define pathological mechanisms.

## Data Availability

Data sharing is not applicable to this article as no datasets were generated or analyzed during the current study.

## Abbreviations

AD: Alzheimer’s disease
ASC: Apoptosis-associated speck-like protein containing a CARD
Aβ: Amyloid-beta
CNS: Central nervous system
COX2: Cyclooxygenase 2
CREB: cAMP-response element binding protein
DβH: Dopamine beta-hydroxylase
HCN: Hyperpolarization-activated cyclic nucleotide-gated
IL: Interleukin
iNOS: Inducible nitrous oxide synthase
LC: Locus coeruleus
L-DOPS: L-threo-3,4-dihydroxyphenylserine
LPS: Lipopolysaccharide
MAPK: Mitogen-activated protein kinase
MCP-1: Monocyte chemotactic protein 1
mPFC: Medial prefrontal cortex
MRI: Magnetic resonance imaging
NE: Norepinephrine
NF-κB: Nuclear factor kappa-B
PET: Positron emission tomography
PET-MRI: Positron emission tomography-magnetic resonance imaging
PFC: Prefrontal cortex
PP: Amyloid precursor protein
TNF-α: Tumor necrosis factor alpha
